# An Assembled Detector Based on Geometrical Constraint for Power Component Recognition

**DOI:** 10.3390/s19163517

**Published:** 2019-08-11

**Authors:** Zheng Ji, Yifan Liao, Li Zheng, Liang Wu, Manzhu Yu, Yanjie Feng

**Affiliations:** 1 School of Remote Sensing and Information Engineering, Wuhan University, Wuhan 430079, China; 2 School of Geodesy and Geomatics, Wuhan University, Wuhan 430079, China; 3 Department of Geography, Pennsylvania State University, State College, PA 16801, USA; 4 Shenzhen Power Supply Planning Design Institute Co., Ltd., Shenzhen 518054, China

**Keywords:** virtual image, vibration damper, assembled detector based on the geometrical constraint (ADGC), object detection, deep learning

## Abstract

The intelligent inspection of power lines and other difficult-to-access structures and facilities has been greatly enhanced by the use of Unmanned Aerial Vehicles (UAVs), which allow inspection in a safe, efficient, and high-quality fashion. This paper analyzes the characteristics of a scene containing power equipment and the operation mode of UAVs. A low-cost virtual scene is created, and a training sample for the power-line components is generated quickly. Taking a vibration-damper as the main object, an assembled detector based on geometrical constraint (ADGC) is proposed and is used to analyze the virtual dataset. The geometric positional relationship is used as the constraint, and the Faster Region with Convolutional Neural Network (R-CNN), Deformable Part Model (DPM), and Haar cascade classifiers are combined, which allows the features of different classifiers, such as contour, shape, and texture to be fully used. By combining the characteristics of virtual data and real data using UAV images, the power components are detected by the ADGC. The result produced by the detector with relatively good performance can help expand the training set and achieve a better detection model. Moreover, this method can be smoothly transferred to other power-line facilities and other power-line scenarios.

## 1. Introduction

The detection of power equipment is critical in power grids, as the safe and effective operation of power directly affects the development of a country’s economy. Due to long-term exposure to the natural environment, power equipment is subjected to severe winds, torrential rains, floods, earthquakes, tree friction, and damage by birds. These external factors accelerate the aging of the power equipment and loose. If the equipment is not checked in a timely manner and repaired immediately, many kinds of problems may result. This situation is akin to a time-bomb on the power grid, the consequences of which are unbearable to contemplate. In order to ensure the safe operation of power transmission and distribution lines, the timely detection of hidden defects in power equipment and the surrounding environment is necessary to provide a basis for line inspection and maintenance and assist in the periodic maintenance of power equipment. Such actions ensure the safe and stable operation of a power grid.

With the recent development of “intelligent” means of power-line inspection, the use of Unmanned Aerial Vehicles (UAVs) to patrol power lines has gradually replaced manual reconnaissance for power towers. Using UAVs to patrol power lines not only removes the limitation of topography and geomorphology, but also greatly improves work efficiency. Additionally, the acquired data can be analyzed offline to make the inspection more comprehensive. Unlike helicopter-based patrolling, there is no need to spend the cost of adjusting machines, stopping, and training pilots. Furthermore, UAVs can patrol power lines quickly. Moreover, UAVs and their airborne equipment are small, light, and easy to carry. Patrol lines are not affected by the region, and are flexible and efficient. The cost of patrol lines is very low, and there is almost no risk to human safety.

In recent years, object detection has benefited from the increasing availability of image data. However, to the authors’ knowledge, there is no public, labeled image dataset for power equipment, due to the strong profession for power application and the narrow usage range. The detection of power equipment has been hampered by a lack of data or the poor labeling of data; therefore, the detection of power equipment is still in a preliminary stage. However, alternatively, virtual data can be easily acquired and automatically annotated, and can serve as perfect training datasets for machine learning.

The purpose of this paper is to accurately detect power equipment using virtual image data when real image data and the corresponding annotation are limited or missing. Due to visual interference, such as electric towers, electric wires, and complex surfaces, the recognition of vibration-dampers has not been well studied. Vibration dampers are typical components of power equipment, and are composed of left and right hammer connectors. In this paper, vibration dampers are considered as the main experimental object. Firstly, engines are generated by general virtual scenes, and the existence of vibration-dampers and some objects that easily obscure the view of vibration-dampers, such as electric towers, are simulated in the scene. Then, a virtual model of a vibration-damper is put into the scene. A virtual sample set, for use as a training sample, is obtained through a certain strategy. An assembled detector based on geometrical constraint (ADGC) is proposed through the virtual dataset. The geometric position relation is used as the constraint, and the Faster Region with Convolutional Neural Network (R-CNN), Deformable Part Model (DPM), and Haar cascade classifiers are combined [1,2,3], which allows the features of different classifiers, such as contour, shape, and texture to be fully used. The result produced by the detector with a relatively good performance can help expand training sets and achieve better detection models.

## 2. Literature Review

With the development of computer graphics, and especially the explosive growth in mobile computing platforms, people can now easily access virtual data using common tools (e.g., Unity3D). In the virtual world, the size and location of virtual models (e.g., vibration-dampers, connectors, and hammers) can be accurately controlled by human beings. Photos can be taken using virtual cameras, and the size and position of the target object can be obtained. Yu et al. [4] divided virtual samples into three types, based on prior knowledge, disturbance thought and distribution function. Previous studies have used datasets for object detection using virtual models. Marin et al. [5] discussed whether the characters in a virtual-reality game scene could be used to train a dataset for the detection of pedestrians. Additionally, a 3D model of a human body was matched with the pose of the characters in the image [6]. The posture of the 3D model was set up by fine-tuning and changing the background to expand the human posture. Furthermore, Aubry et al. [7] used the histogram of oriented gradient (HOG) features and linear discriminant analysis (LDA) based on components for the identification of the class of chairs in an indoor environment with thousands of different shapes of chairs, and estimated the positions of chairs. 

Moreover, Gungor et al. [8] discussed communications technologies and requirements for smart grids. There are many important open research issues for the realization of smart grid communications and applications. Kabalci et al. [9] aimed to clearly define what a smart grid is and what kind of communication methods are used in smart grids. All components of a smart grid are introduced in a logical way to facilitate the understanding, and communication methods are presented regarding their improvements, advantages, and lacking features. Kelly et al. [10] adapted three deep neural network architectures to energy disaggregation, namely (1) a form of recurrent neural network called long short-term memory (LSTM), (2) denoizing autoencoders, and (3) a network which regresses the start time, end time, and average power demand of each appliance activation. Altrabalsi et al. [11] addressed the challenges via a practical low-complexity low-rate NALM by proposing two approaches based on a combination of two machine learning techniques, namely k-means clustering and Support Vector Machine (SVM), exploiting their strengths and addressing their individual weaknesses.

There are two general methods for detecting electric power equipment. One uses an optical image, and the other uses a multi-view geometry for transmission-line reconstruction and recognition. A method combining Scale-Invariant Feature Transform (SIFT) and Random Sample Consensus (RANSAC) has been proposed to analyze images of electric power equipment and detect anomalies in such equipment using variable parameters [12]. Li et al. [13] used a coupled-pulse neural filter to remove the background noise and obtain the edge of an image. Additionally, the improved Hof transform method has been used to extract transmission lines from images. With the development of sensor hardware technology, it has become popular to use different types of sensors to judge the safety of transmission-line environments. Jwa et al. [14] proposed a segmented line-detection method based on voxel analysis, which realizes the 3D reconstruction of transmission lines using an airborne LIDAR point cloud.

To the authors’ knowledge, research on detecting power equipment through virtual models and machine learning is still at a preliminary stage. Zhai et al. [15] proposed a method based on a training Haar cascade classifier using a 3D model of an insulator for detection. Zhai et al. [16] determined the location of insulators by the registration of a 3D tower frame and an image of the tower frame. Combined with the earth moving distance, the insulators’ self-explosion was detected. Object detection based on machine learning mainly includes the following methods. Viola et al. [3] proposed a cascade classifier based on Haar features trained by the AdaBoost algorithm for face recognition and gesture recognition, which was used in many successful applications. Dalal et al. [17] firstly proposed HOG features, and learned an object’s gradient model using SVM to complete the recognition, winning the PASCAL target recognition challenge in 2006. The DPM method [18] has been improved based on HOG features and has a wide range of applications in deformable objects, such as pedestrian and human posture. Comparatively power equipment are mostly rigid objects. However, power equipment is normally a kind of combination of different parts and has different sizes, specifications and composite structure. For similar deformable conditions, the DPM method here is also adapted for power equipment detection. With the recent extensive application of convolutional neural networks (CNNs) in deep learning, great progress has been made in target detection. Girshick et al. [18] proposed a detection method based on local image regions and CNN feature extraction and classification. Girshick et al. [19] and Ren et al. [1] performed optimization for regional feature extraction and detection region generation, respectively, which were accelerated by sharing weights with CNN classification. 

## 3. Methodology

This section describes the target-detection methods based on machine learning—including Faster R-CNN, DPM, and AdaBoost training based on Haar features—which were used in the present study. Taking a vibration-damper as the main detection object, the ADGC is proposed using a virtual dataset. The geometric position relation is used as the constraint, and the Faster R-CNN, DPM, and Haar cascade classifiers are combined, which allows the features of different classifiers, such as contour, shape, and texture to be fully used in order to obtain more accurate detection results.

### 3.1. Construction of Virtual Dataset

#### 3.1.1. Reconstruction of Virtual Objects and Scenes Using a Virtual Engine

The virtual image data used in the present study is mainly composed of two parts: One is the general scenario of virtual objects; the other consists of virtual trained objects to be identified and supporting devices. Virtual image data should restore the real environment. The general virtual scene includes terrain, grass, houses, sky, etc., which are available as plug-ins in the Asset Store of the Unity3D platform. Considering the development cost of the virtual scene, and in order to give a realistic scene, the virtual scene in this paper is a suburban scene with low buildings, grass, and scattered stones on the ground. The virtual training objects to be identified and the supporting devices are the key components for the virtual experimental data. In this paper, the object to be trained and identified is a vibration-damper, while the supporting devices include a high-voltage tower and an electric wire. To ensure that the shapes of the devices were as realistic as possible, all of these devices were manually modeled using physical parameters and profiles in the 3DS Max software. The sizes of the devices can be adjusted and controlled by the virtual scene, which ensures that the virtual object has a proper proportional relationship with the virtual scene. The model attitude and quantity can also be adjusted by the demand of different data models. The vibration-dampers modeled in the experiment were the two most common types in actual working.

#### 3.1.2. Virtual Sample Data Generation

**Figure 1:**
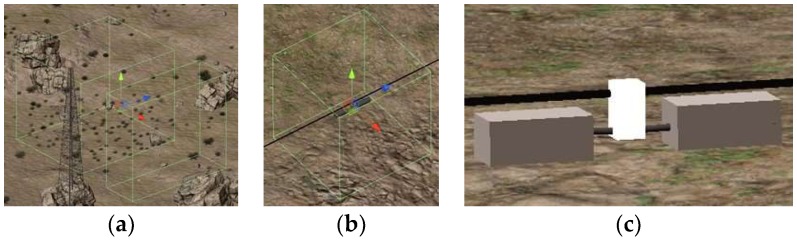
Virtual scenario processing: (**a**) Camera movement area; (**b**) movement area of the photography center; (**c**) the smallest outer rectangular body.

The acquisition of virtual image data is based on the function of cameras in a game engine. The game engine simulates and photographs the objects—i.e., vibration-dampers in this experiment—and outputs the image in a common image format (usually jpeg). In the process of obtaining virtual images, the following points are considered:

1. Ensure training effectiveness. The virtual images are rendered by various condition parameters, such as camera positions and orientations. According to the scene structure, a few inter-visible blocks are set up for the object and the camera to obtain a good camera setting area. The camera moves randomly in the preset area based on a random algorithm to ensure the effectiveness and diversity of the virtual image data. As shown in Figure 1a, two cuboidal regions are determined as random motion areas for the camera;
2. Ensure the diversity of the object imaging angle. The camera should not be fixed to a center point for shooting. Instead, a rectangular or square area is preset at the center of the object, and the center point of the camera moves randomly in the preset area based on a random algorithm, as shown in Figure 1b;
3. Reduce the workload of manual annotation. A minimum external cuboid can be preset to the structure of interest in the virtual scene, where the precise coordinates of the eight vertices can be obtained by the transformed attribute of Unity3D. The camera exterior orientation elements in real-time can also be obtained in the same way. According to the image coordinates of the eight vertices of the smallest cuboid, the smallest outer rectangle of the object and the object parts are obtained, as shown in Figure 1c;
4. Before taking a photo, it should be determined whether the object is in the image. The maximum and minimum coordinates of the outsourced object enclosure in the image coordinate system are calculated in real-time by the smallest outer rectangle of the object. By comparing the maximum and minimum coordinates of the object bounding box in the image coordinate system with the image resolution, it can be determined whether the image has gone beyond;
5. In the 3D virtual game engine, an event is usually carried out in a frame. After completing the aforementioned steps 1–3, the corresponding function is executed by frame, and an image is generated for each frame.

**Figure 2:**
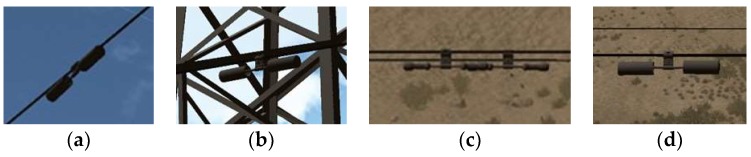
Virtual Samples: (**a**) Sky background; (**b**) tower background; (**c**) overlapping vibration-dampers; (**d**) ground background.

In this study, a total of 7062 images were generated for the vibration-damper samples using the above method, including 3529 images of the FD vibration-damper and 3533 images of the FR vibration-damper, as shown in Figure 2.

### 3.2. Object Detection Based on Machine Learning 

#### 3.2.1. Object Detection Operator

Taking the virtual vibration-damper data as a training set, a detection model was designed based on geometric constraints with three detection classifiers, namely (1) Faster R-CNN [1], which is based on a deep CNN network, (2) DPM [2], which is based on HOG features and latent SVM, and (3) the cascade classifier [3], which is based on AdaBoost training for Haar features.

Faster R-CNN is a multi-layer deep network for object detection. It includes a shared weight layer and two parallel networks, namely a region proposal network (RPN) and the object detection network (Fast R-CNN). The RPN provides a candidate region for object detection in the Fast R-CNN network. Fast R-CNN can also be divided into two parallel external frames, namely a regression network and an object class scores network. Then, the network output is the location coordinates and probability scores for the vibration-dampers to be inside the detected images. 

DPM is similar to the Haar cascade classifier, which firstly extracts features by the specified method and then trains them to generate the classifier. DPM obtains the contour information of an object by extracting the HOG features and establishes the relative positional relationships between the object and the components to some extent so as to detect the object. Without the vibration-damper parts marked, the HOG feature of the vibration-damper is obtained respectively by images with two different resolutions, representing different scales of the vibration damper. The positional relationship between the vibration-damper model and each sub-model is obtained through latent-SVM learning. In the detection stage, it can be determined whether a region contains a vibration-damper by the positional relationships between the models and sub-models, and the possibility score and the external rectangle of the vibration-damper can be calculated.

The locations of the object and its components can be obtained accurately using the image of the virtual simulation scene. When training the cascade classifier Haar features, three cascading classifiers can be established, namely for the entire vibration-damper, the connector, and the vibration-damper on both sides, respectively. Comparatively, when training the classifier with AdaBoost, square samples as input dataset with uniform size range are made for cascade classifier. The output of cascaded classifiers based on Haar features are the entire vibration-damper, the connector, and the outer square of the vibration-damper body. However, these classifiers do not obtain good classification results, due to the low number of features.

In the above object detection methods based on machine learning, the Haar features represent the contrast characteristics of light and shade between two or more rectangular regions in the image. The detection based on the HOG feature obtains object contour information using image gradient learning. The detection based on a depth network achieves the automatic induction and fitting of the object features through learning, which allows a fast detection speed and high accuracy. However, for virtual images, it is often difficult to simulate all completely realistic object’s model; modelers can only model based on physical references and experience, and are limited in terms of model styles, especially texture mapping styles.

#### 3.2.2. Assembled Detector Based on the Geometrical Constraint (ADGC)

**Figure 3:**
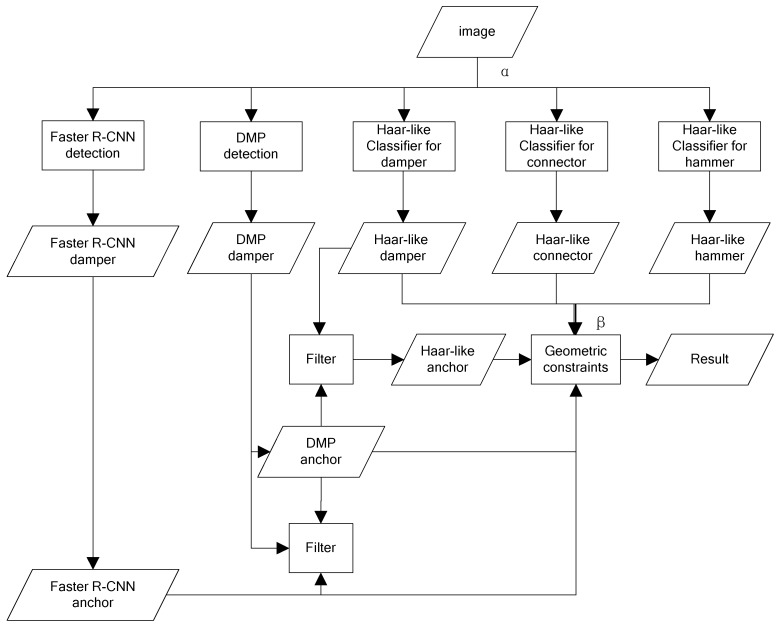
Classifier combination flow chart.

Based on the different characteristics of the classifiers, ADGC is proposed in this paper. The premise of ADGC is the results of multi-resolution detection of each classifier. Based on the detection results, buffer regions (anchors) are selected in turn according to the theoretical confidence of each classifier, and the nearby detection results of other classifiers are also extracted. By comparing the sizes, locations, and scores of the buffer regions with those of the classifier-detected regions, it is possible to determine whether to use the candidate region as the target object. Taking the vibration-damper as an example, the whole process of ADGC is shown in Figure 3.

Faster R-CNN with higher complex and hierarchical parameter structures is used to detect the virtual vibration-damper, and therefore has a higher degree of confidence. However, without real data, there is a limitation in transfer learning for Faster R-CNN. The DPM classifier has a higher degree of confidence for the combination of the whole vibration-damper and part of the vibration-damper. It represents the contour information of the vibration-damper, and the detection result based on DPM is, thus, affected when there is no obvious change in gradient between the vibration-damper or some part of the vibration-damper and the background in the actual data. The single-class Haar classifier is affected by less-effective features and no rich feature expression. When using the single-class Harr classifier, detection is greatly disturbed by the complex background, especially in the classification of connectors and components. The contrast between light and shade is poor in the available rectangular block. Then, there will be a larger error in the detection. In ADGC, the order of the application of the three classifiers as anchors is as follows: Faster R-CNN, DPM, and class Haar. The specific process is as follows: First, take the detection result using Faster R-CNN as an anchor, evaluate the detection results of the DPM near anchor in turn, and give a score by comparing their width and distance. This process is given by

(1) $$S_{DPM}=\underset{i}{\max}w_{1}f(\frac{W_{i}-W_{a}}{W_{a}})+w_{2}f(\frac{H_{i}-H_{a}}{W_{a}})+w_{3}f(\frac{d_{i}}{W_{a}})+DPM_{i},\;$$

where $W_{i}$ and $H_{i}$ are the width and height of the current DPM detection rectangle, respectively, $W_{a}$ and $H_{a}$ are the width and height of the current anchor, respectively, $d_{i}$ is the Euclidean distance from the center of the DPM rectangle to the current anchor center, and $DPM_{i}$ is the score of the current DPM detection rectangle. The function f(x), which is defined in Equation (2), is a Gaussian function. The parameters $w_{1}$, $w_{2}$ and $w_{3}$ are all weights. Here, $w_{1}=0.25,w_{2}=0.25,w_{3}=0.5$. The average weighted with the center distance is the geometric score of the current DPM detection results, and the maximum score is 1:

(2) $$f(x)=e^{-\pi x^{2}}$$

The next step is to evaluate the representation of the Haar in the anchor. The Haar classifier can be divided into the whole vibration-damper, the connector, and three parts, where the positions of the vibration-damper body and connector are different for different positions of the vibration-damper. Then, the three ideal positions of the vibration-damper are respectively defined as 20° rotation to the right, 20° rotation to the left, and no rotation. There are six positions of the vibration-damper body in the three ideal positions of the vibration-damper body, and the position is selected from the nearest ideal vibration-damper position. As the six vibration-damper positions are divided into three positions, the final vibration-damper body takes the highest score:

(3) $$S_{hammer}=\overset{3}{\underset{i=1}{\max}}\underset{j}{[\max}w_{1}f(\frac{W_{j}-W_{a}}{W_{a}})+w_{2}f(\frac{d_{j}}{W_{a}}){]}_{2i-1}+\underset{j}{[\max}w_{1}f(\frac{W_{j}-W_{a}}{W_{a}})+w_{2}f(\frac{d_{j}}{W_{a}}){]}_{2i},\;$$

where $w_{1}=0.5,w_{2}=0.5$, and $d_{j}$ represents the distance between the center of the current vibration-damper body and the corresponding ideal center of the vibration-damper body. The other terms are the same as in Equation (1). 

The Haar classifier outputs a square box, so the shape evaluation only takes the width value. When the weight of the vibration-damper is obtained, an approximate position of the vibration-damper in the anchor can be also obtained. Then, the ideal position of the connector is calculated using the position of the connector. Equation (4) is similar to Equation (1):

(4) $$S_{bridge}=\underset{i}{\max}w_{1}f(\frac{W_{i}-W_{a}}{W_{a}})+w_{2}f(\frac{d_{i}}{W_{a}}),\;$$

where $d_{i}$ is the distance between the current connector center and the corresponding connector center in the ideal vibration-damper body evaluated by the vibration-damper body. The weight value is the same as in Equation (3).

Based on the evaluated position of the vibration-damper body, the ratio of the height and width of the corresponding ideal vibration-damper can be obtained when evaluating the overall classifier of the vibration-damper by the Haar feature. Then, the theoretical height of the square area determined by the Haar classifier can be obtained through the ratio of the height and width. Thus, the height and width can be calculated, as shown in the following Equation (5):

(5) $$S_{haar}=\underset{i}{\max}w_{1}f(\frac{W_{i}-W_{a}}{W_{a}})+w_{2}f(\frac{r_{t}W_{i}-H_{a}}{W_{a}})+w_{3}f(\frac{d_{i}}{W_{a}}),\;$$

where $r_{t}$ is the ratio of the height and width in the corresponding position for the vibration-damper. The other items are the same as in Equation (1).

Finally, the final score of the anchor is calculated by combining the scores evaluated by each classifier, as shown in the following Equation (6):

(6) $$S_{T}=w_{1}S_{F}+w_{2}S_{DPM}+w_{3}S_{haar}+w_{4}S_{bridge}+w_{5}S_{damper},\;$$

where $S_{F}$ is the value using Faster R-CNN at the anchors. The weights in the equation are taken as $w_{1}=1,w_{2}=1,w_{3}=0.9,w_{4}=0.8,w_{5}=0.7$ empirically.

After all Faster R-CNN detection areas have been anchored, the area calculated by DPM is calculated and evaluated as a new anchor, which is not calculated in the Faster R-CNN anchor. The evaluation process is basically the same as for the Faster R-CNN; the difference is that the first item ($S_{F}$) is not calculated in Equation (6) as its value is zero, while the second item ($S_{DPM}$) contains only the DPM score of the anchor. After making an anchor for the remaining DPM, the vibration-damper overall classifier of the Haar feature is calculated in the same way as the anchor, which is not involved in the first two steps of the evaluation calculation. The cascade classifier itself has no score, and the corresponding Equation (6), therefore, only holds the last two items here.

## 4. Experimental Results and Discussion

This experiment was divided into two parts. The first part was the generation of the virtual scenes and the virtual samples, focusing on the vibration-damper and the surrounding environment. In the second part, the virtual data generated in the first part was trained to generate the classifier. Then, the ADGC method was applied to detect the power equipment. 

### 4.1. Generation of Virtual Sample

**Figure 4:**
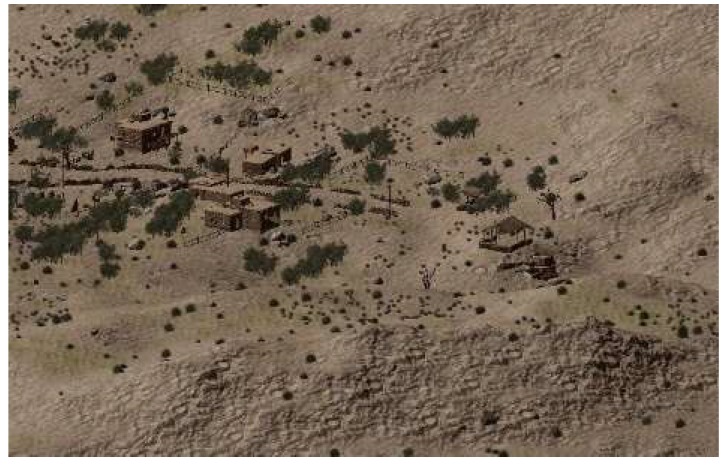
The basic virtual scene.

**Figure 5:**
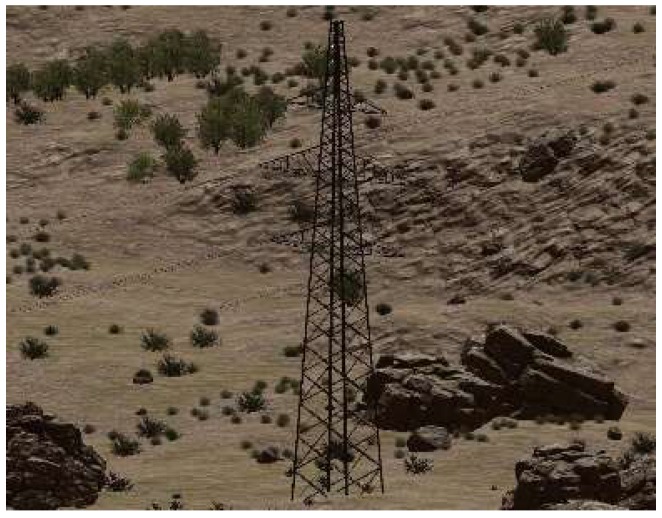
The imported electric tower.

This experiment aimed to build a virtual scene of a common field scene for the inspection of electric power, which usually includes sky, land, rocks, grass, and houses. The Middle-East Environment scene in the Unity3D material stores is similar to the experimental environment. Then, some material was bought from the store to form the basis of the virtual scene, which was then imported to Unity3D, as shown in Figure 4. In the virtual power scene, the main background of the vibration-damper includes an electric tower and a power line, in addition to the natural environment. The tower was modeled artificially in the 3DS Max software and then imported into the virtual scene. According to the relationship between the proportions of the tower and the other objects in the scene, the height of the tower in the scene was set to 59 unit lengths, as shown in Figure 5.

**Figure 6:**
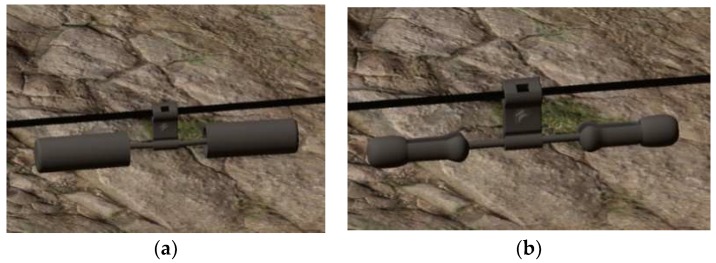
The two types of vibration-dampers. (**a**) FD type; (**b**) FR type.

The vibration-damper, which is the main target of this experiment, was also modeled in the 3DS Max software. 3D models of an FD type and an FR type vibration-damper were built. If there is a power line, there is a vibration damper. The structure of the power line is relatively simple, and it takes the base cylinder model in Unity3D as the electric wire and uses the black material. There are two types of vibration-dampers shown in Figure 6, which are called FD type and FR type respectively in the field of power profession.

After importing the vibration-damper into the virtual scene, the relative position of the vibration-damper and the electric tower was considered. First, the wire was pulled out from the wire connection on the electric tower, which is parallel to the ground and connected to the vibration-damper. The distance between the vibration-damper and the tower was set to be about 6 unit lengths. In Unity3D, two cuboidal frames were prepared parallel to the power line to simulate the possible moving range for the virtual camera. The length, width, and height of the two cuboidal frames were respectively 33.40278, 32.40397, and 39.21832, and 13.41675, 34.09087, and 39.21832. The distances between the centers of the two cuboids and the center of the vibration-damper were respectively 16 and 23 unit lengths. The random moving range of the focus center of the virtual camera was centered on the vibration-damper, and its length, width, and height were all 3 unit lengths. In order to identify the vibration-damper correctly for their overlap, a case with two power lines close to the vibration-dampers was also built. The other parameter settings were the same as above.

**Figure 7:**
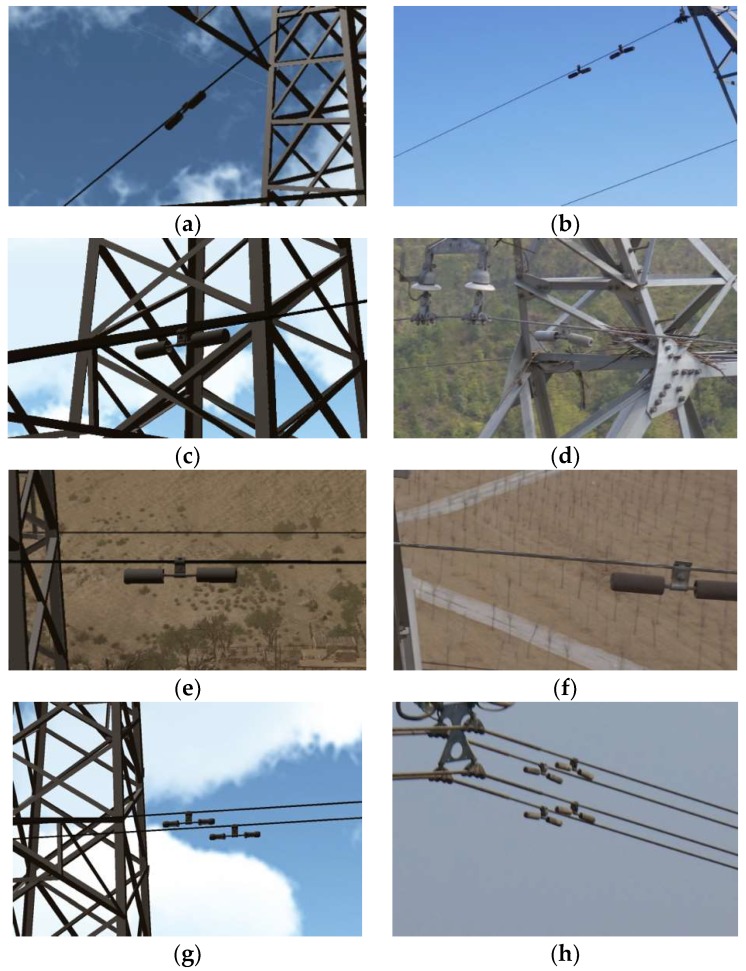
Comparison of real (right-hand column) and virtual (left-hand column) images of vibration-dampers. (**a**,**b**) Sky background; (**c**,**d**) tower background; (**e**,**f**) ground background; (**g**,**h**) overlapping dampers.

There were a total of 7062 sample images and their corresponding annotation files, including 3529 images of the FD type vibration-damper and 3533 images of the FR type vibration-damper. The images cover a variety of photographing positions and reasonable shooting distances. The background includes the sky, the tower, the ground, a house, and a mixture of various backgrounds. In addition to the single vibration-damper model, there were 1020 images in which the vibration-damper overlapped with another object. Figure 7 shows a comparison of real and virtual images of the two types of vibration-damper.

### 4.2. Virtual Sample Experiment

Three classifiers were used in the experiment: The Faster R-CNN classifier, the DPM classifier, and a cascade classifier based on Haar characteristics. The cascade classifier based on Haar features had the whole vibration-damper classifier, the connector classifier, and the vibration-damper body classifier, respectively. The DPM classifier and the Haar cascade classifier were trained by a single thread respectively using an Intel Core i7-6800k and an AMD Phenom™ II X6 1090T CPU computing platform. The Faster R-CNN classifier was trained using the Nvidia GTX1080 GPU platform. 

#### 4.2.1. Faster R-CNN Training

**Figure 8:**
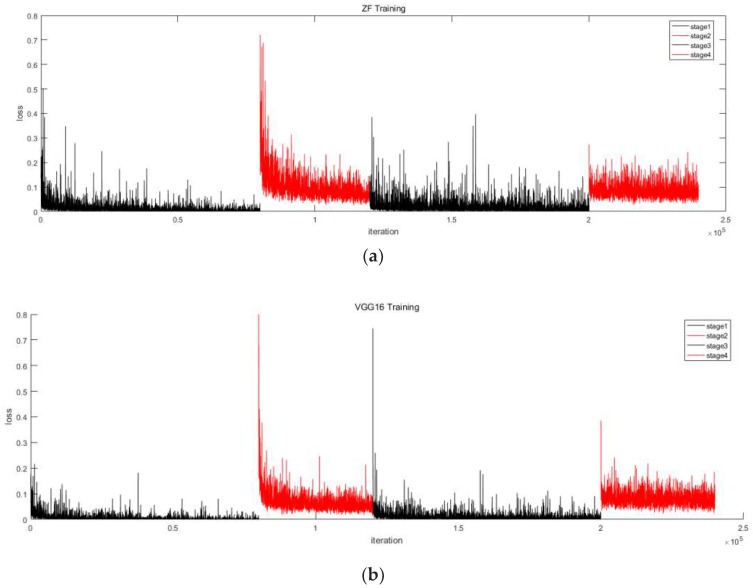
The loss value of Faster R-CNN training. (**a**) Using the ZF-net. (**b**) using the VGG16-net.

We implemented the Faster R-CNN classifier in the Caffe deep learning framework, as described in [1]. Two CNN models were used in the shared weight layer as a contrast test. The first was a CNN model proposed by Zeiler and Fergus [20], called the ZF-net. The ZF-net has seven network layers, not counting the input and output layers. The first five layers are the convolution layers, and the last two layers are the fully connected layers. In this study, the first five layers are as the shared weight value. The second CNN model was a 16-layer convolution network proposed by Simonyan and Zisserman [21], called the VGG16-net. In this study, the Faster R-CNN shared weight layer was the first 13 layers, i.e., all layers except for the fully connected layers (the last three layers). These two kinds of the network were used to train parameters on ImageNet in order to initialize the network. The training procedure was inspired by the four steps described in [1]. The first step is to train an RPN with the initial value of ImageNet; the second step is to train a Fast R-CNN detection network with the initial value of ImageNet; the third step is to fix the shared weight layer which is obtained in the second step and to tune the RPN; and the fourth step is to fix the shared weight layer and tune the Fast R-CNN network. The numbers of iterations for these four steps are 80,000, 40,000, 80,000, and 40,000, respectively. The training time for VGG16 is 662 min 43 s and that for ZF is 297 min 21 s. As shown in Figure 8, during training, the VGG16 network has a smaller loss value than the ZF network.

#### 4.2.2. DPM Training

**Figure 9:**
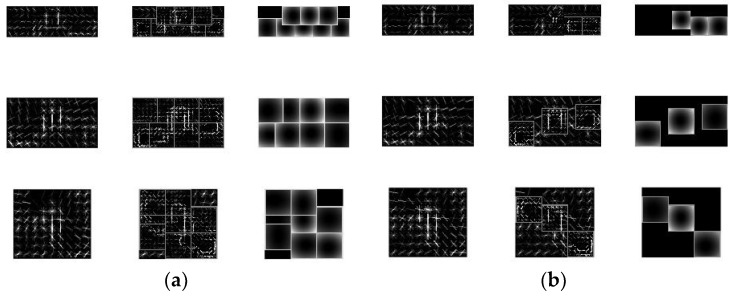
Histogram of oriented gradient (HOG) features trained by DPM. (**a**) Eight sub-models; (**b**) Three sub-models.

The training of the DPM classifier was implemented using the Matlab (MathWorks, Natick, MA, USA) toolset provided by Girshick et al. [22]. A total of 7062 virtual images were trained for the positive samples (i.e., images which contained a vibration-damper). There were no negative samples (i.e., images which did not contain a vibration-damper). In the training, a sufficient number of negative sample images can be generated at different resolutions using the provided negative samples. The preparation of the negative samples does not require much work for the actual scene. There are 50 negative sample images, ranging in size from 15 million to 24 million pixels. In these images, the vibration-damper may appear in the sky, over the ground, and other electrical equipment may also appear. For the DPM classifier, two classifiers were trained for comparison. Taking into account the relative rotation of the cameras, each classifier had three models, while the number of sub-models was three and eight. The DPM classifier with eight sub-models was trained for 4830 min 43 s, while the classifier with three sub-models was trained for 2510 min 33 s. As shown in Figure 9, the result of the training is HOG models, in which the feature relationship among the vibration-damper components is expressed well by the classifier with three sub-models, and is similar to the prior knowledge regarding the vibration-damper. However, the eight sub-models are redundant.

The training of the Haar cascade classifier was based on the training method of the AdaBoost cascade classifier provided in OpenCV. Since the virtual images provide the position of the whole vibration-damper and its components, the positive samples are all the objects extracted from the 7062 virtual images for the whole vibration-damper and the connector of the vibration-damper. The process of acquiring the negative sample with the Haar cascade classifier is similar to that with the DPM classifier. As each vibration-damper contains two vibration-damper bodies, in order to avoid making the sample too large and the training time too long, 4000 virtual vibration-damper images were randomly selected from the virtual sample, 8000 vibration-damper bodies were used as the positive samples for the vibration-damper classifier, and the negative samples were the same as in Section 4.2.2. The samples obtained using the three classifiers were normalized to 20 × 20. The number of cascaded layers for the strong classifier was 20. The lowest correct rate of each layer was set to 0.999. The maximum false classification rate of the negative samples was set to 0.5. The final training time of the whole classifier was 13,379 min 59 s, in which the 20-level strong classifier used 2064 features. The training time for the connector classifier was 7188 min 34 s, and the 20-level strong classifier used 1181 features. The training time for the vibration-damper body classifier was 13,139 min 29 s, and the 20-level strong classifier used 1623 features.

#### 4.2.3. Detection and Analysis Using ADGC

The trained classifier was applied to real images with different resolutions to detect vibration-dampers. Moreover, these detection results were then combined by the ADGC method to get the final detection results.

The real image dataset contained 19 images with power scenes and vibration-dampers, which were not inside the training set. The images contained 88 vibration-dampers, which were identified by human inspection or deduced from the scene context. The vibration-dampers were divided into two types in the actual scenario, namely foreground and background. Foreground vibration-dampers refer to vibration-dampers, which are the subject of shooting. A total of 58 vibration-dampers were clearly visible in the images. Vibration-dampers that are close to the camera have no occlusion or only a small amount of occlusion. There was a total of 30 background vibration-dampers in the images, which had small margins or were obscured by other electric power equipment. If the Intersection over Union (IoU) was larger than 0.5, the vibration-damper was regarded to have been correctly detected. 

**Figure 10:**
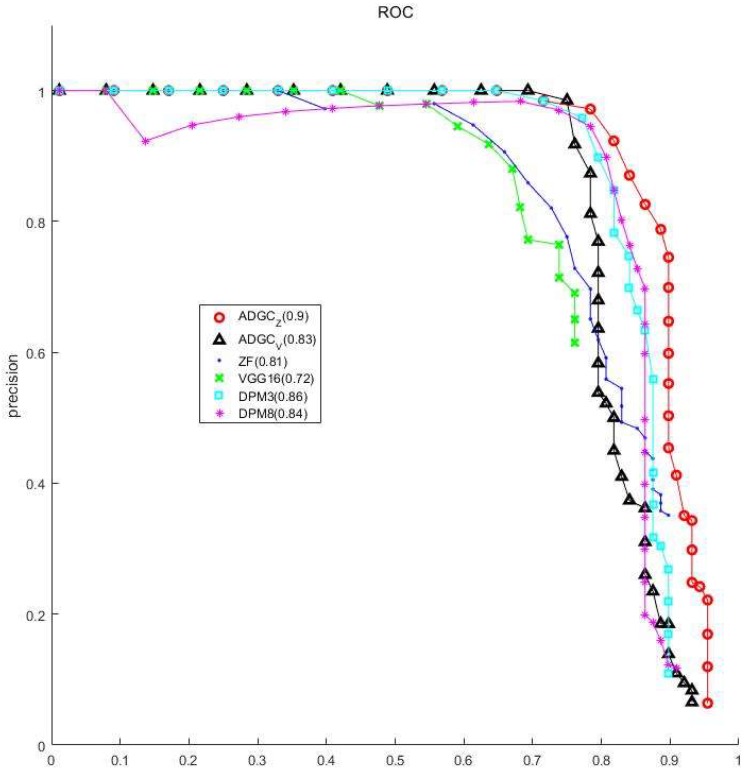
Receiver operating characteristic (ROC) curves for the virtual samples.

The values highlighted in bold in Table 1 are the best results. The detection result of ADGC (ZF) is the best, with 84 vibration-dampers being detected—58 foreground vibration-dampers and 26 background vibration-dampers—while 26 background vibration-dampers were detected by ADGC (VGG16) and ZF. Compared with the ZF-net, VGG16-net has a deeper network with a lower loss value in training for the Faster R-CNN classifier; however, a lower Average Precision (AP) was obtained for the foreground and background vibration-dampers. The VGG16-net has a stronger fitting ability and a more accurate learning for the training set, however, the detection result was not as good as that for the ZF-Net, due to a data domain bias between the virtual samples, as the training set, and the real samples, as well as overfitting for the training domain. Figure 10 shows Receiver Operating Characteristic (ROC) curves for the real samples, in which ADGC_Z_ and ADGC_V_ represent the methods of the ZF-Net and the VGG16 network, and the three sub-models were all used in the DPM classifier. As shown in Figure 10, the larger the area of the curve, the better the algorithm is. From Table 1 and Figure 10, it can be seen that the ADGC based on the VGG16 network has a relatively low AP for the less detected vibration-dampers, but has higher values of ROC.

**Figure 11:**
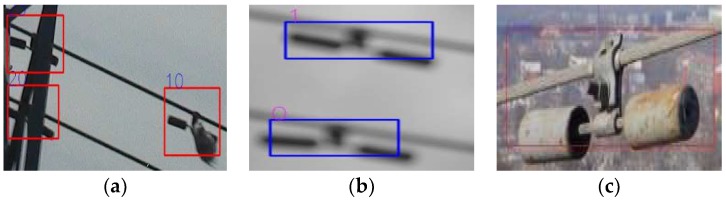
Detection results for difficult samples. (**a**) Obscured vibration-dampers; (**b**) fuzzy vibration-dampers; (**c**) vibration-dampers with color variation.

As shown in Figure 11a,b, the Faster R-CNN classifier based on the ZF-net has a better detection ability for obscured and fuzzy vibration-dampers, which is rarely seen in virtual samples. The two classifiers showed some generalization ability, i.e., both detected the colorless vibration-dampers in the virtual samples, as shown in Figure 11c. Additionally, the ZF-net with fewer layers also have higher efficiency. It takes 0.08 s for an image with a size of 1 million pixels to be detected by the ZF-Net, whereas it takes 0.18 s using VGG16 under the same conditions. 

**Figure 12:**
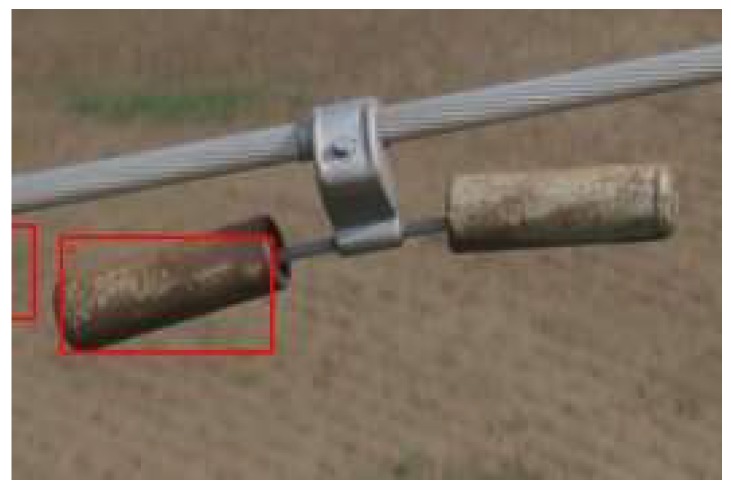
Detection result for components similar to the background obtained using DPM.

Regarding the two DPM classifiers, the classifier with three sub-models obtained a better result than the one with eight sub-models. When the color contour of the vibration-damper is similar to that of the background, the recognition by the DPM classifier based on HOG feature becomes unstable. Then, the DPM classifier needs to combine other classifiers, as shown in Figure 12. The DPM classifier with three sub-models has an obvious advantage in terms of speed. While the detection time achieved with the DPM classifier with eight sub-models was 1.29 s, that achieved with the DPM classifier with three sub-models was about 0.72 s, i.e., more than 40% less. Therefore, selecting the appropriate number of models and the number of sub-models for the DPM classifier based on prior knowledge can obtain better results. For training classifiers for other electrical equipment, it is recommendable to make reasonable use of prior shooting and structural information about power equipment, and to decompose the complex structure of the power equipment appropriately. Better results will be obtained with a combination of the DPM classifier with Haar classifier training.

## 5. Conclusions

Due to the lack of images of power equipment and reliable annotation data, in this study, a low-cost virtual scene of a power line was created, and training samples for power-line components were generated in a cost-effective way. The method of generating annotation was based on a virtual scene development framework conducted using the Unity3D platform. Taking a vibration-damper as the main object, the assembled detector based on the geometrical constraint (ADGC) was proposed and was used to analyze the virtual dataset. The geometric positional relationship was used as the constraint, and the Faster R-CNN, DPM and Haar cascade classifiers were combined, which provided the advantages of the features of the different classifiers, such as contour, shape, and texture. The result produced by the detector with a relatively good performance can help to expand the training set and achieve a better detection model. Moreover, this method can be smoothly transferred to other power-line facilities and other power-line scenarios.

## Figures and Tables

**Table 1:** Detection results for real images using the virtual dataset as the training sample.

		Average Precision (AP)	Result
VGG16	Foreground vibration-damper	0.858111	52
	Background vibration-damper	0.420954	15
	TOTAL	0.729443	67
ZF	Foreground vibration-damper	0.881037	54
	background vibration-damper	0.512678	26
	TOTAL	0.812168	80
DPM 8-part	Foreground vibration-damper	0.906523	55
	Background vibration-damper	0.584122	25
	TOTAL	0.835118	80
DPM 3-part	Foreground vibration-damper	0.950293	56
	Background vibration-damper	0.609376	23
	TOTAL	0.857494	79
ADGC (VGG16)	Foreground vibration-damper	0.945191	56
	Background vibration-damper	0.518371	26
	TOTAL	0.829437	82
ADGC (ZF)	Foreground vibration-damper	0.952389	58
	Background vibration-damper	0.709812	26
	TOTAL	0.899569	84
